# SSELF: A Specific SEmiautomated Lifecycle Footprinting framework to go beyond generic data in LCA

**DOI:** 10.1111/jiec.70056

**Published:** 2025-06-25

**Authors:** Marit Salome Rognan, Manuele Margni, Guillaume Majeau-Bettez

**Affiliations:** 1 Department of Chemical Engineering, CIRAIG, École Polytechnique de Montréal, Montréal, Quebec Canada; 2 Department of Mathematics and Industrial Engineering, CIRAIG, École Polytechnique de Montréal, Montréal, Quebec Canada; 3 Institute of Sustainable Energy, University of Applied Sciences and Arts Western Switzerland (HES-SO), Sion, Switzerland

**Keywords:** big data, carbon footprint, climate change, industrial ecology, life cycle assessment, supply chain data

## Abstract

Advancements in life cycle assessment (LCA) and environmentally extended input–output analysis enable quick generic estimations of the environmental footprint of almost any product and service. However, going beyond a generic estimate to an assessment based on actual, specific supply chain data remains costly and impracticable and demands significant sharing of proprietary data between supply chain actors and the LCA practitioner. Achieving widespread specificity in LCA requires fundamentally changing the way inventory and emission data are collected, stored, and exchanged. This research develops the SSELF (Specific SEmiautomated Lifecycle Footprinting) framework to go beyond generic data in LCA in a way that can scale up, while safeguarding sensitive data. A key feature of the framework is decentralizing inventory collection and footprint calculations. Thus, production functions remain private and upstream impacts are calculated using an iterative approach with a database of unique product identifiers and the footprints reported by other users, capturing changes in the footprints of suppliers. Although this substantially reduces the effort of footprint assessments, implementing the framework in practice presents new challenges, which are identified and discussed in this paper along with recommendations on how they can be addressed and their implications. This work provides important insight into how to get to a point where every product and service has its unique footprint. Broad access to footprints with more specificity is necessary to help consumers reduce their consumption-based impacts and make companies take accountability for, and reduce, their indirect impacts.

**Supplementary Information:**

The online version of this article (doi:10.1111/jiec.70056) contains supplementary material, which is available to authorized users.

## INTRODUCTION

### Background and motivation

Life cycle assessment (LCA) and environmentally extended input–output analysis (EEIO) have emerged as valuable tools to assess the environmental impact of products and services (Guinee et al., 2011; Kitzes, 2013). They enable the estimation of environmental footprints of nearly any system and allow for comparative assessments. However, the usefulness and reliability of their results heavily depend on the quality, specificity, and completeness of the data used.

**FIGURE 1:**
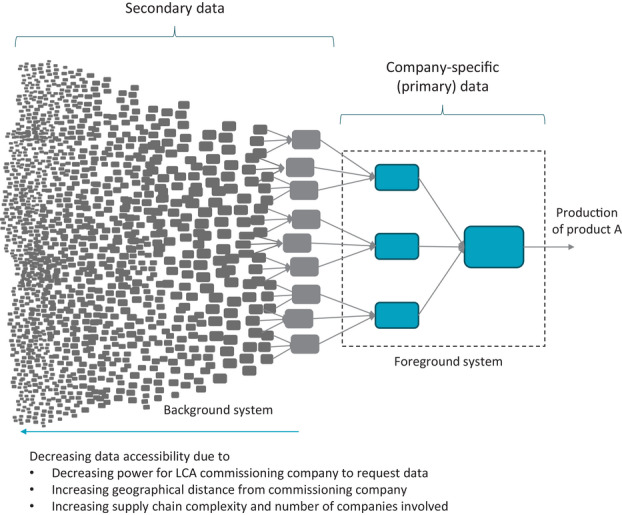
Data collection challenges in a typical process tree in life cycle assessment (LCA).

One significant challenge in LCA is the scarcity of primary data for processes outside the walls of the LCA-commissioning company, which represents the foreground system in Figure 1. This leads to the use of generic, truncated, and at times outdated secondary data to model the much larger background system (Miah et al., 2018; Zargar et al., 2022), which in most cases holds the largest share of the cradle-to-gate impacts (CDP, 2022). Consequently, there can be large uncertainties and potential biases in LCA results, particularly for systems that are more complex or heterogeneous, and in which the variability of the background system is a significant factor for the resulting impact of the product system (Cox et al., 2018; Häfliger et al., 2017). As illustrated in Figure 1, the further upstream from the foreground system, the more challenging it becomes to collect primary data. Furthermore, the strive for data openness and transparency poses scalability challenges in LCA (Schaltegger & Csutora, 2012). Although LCA databases aim for broad coverage, achieving this hinges on companies sharing disaggregated foreground data. However, the sharing of such data is hindered by concerns about reputation, industrial secrets, and competitive advantage (Ciroth et al., 2019), making an openly accessible disaggregated company-specific database unlikely.

In a setting where a corporation has thousands of suppliers and each of them has; numerous suppliers as well, the feasibility of expanding the foreground system beyond one's own processes becomes very low. Although the availability of carbon footprint data in particular is growing with a heightened climate change awareness, analysts will hesitate to use carbon footprints calculated by other parties due to the lack of harmonization in assumptions, system boundaries, and inevitable proxy choices. Thus, their credibility and usefulness are undermined by methods and modeling inconsistencies that stem from lack of access to system-specific primary data (Bolton & Kacperczyk, 2021; Downie & Stubbs, 2012; Rondoni & Grasso, 2021; Stenzel & Waichman, 2023; TCFD, 2022). Not to mention, while we can expand the foreground system with some research and collaboration effort, there always comes a point where the practitioner has to turn to generic processes from databases such as ecoinvent (Wernet et al., 2016).

These challenges hinder the creation of a comprehensive and consistent database that can effectively integrate analyses, moving beyond reliance on proxy data alone. Without complete industry transparency, which is unlikely to be achieved in the near future, our ability to accumulate knowledge is hindered. In mathematical terms, with the current approach, there is a need for a single consistent technology matrix, either in an aggregated database (such as GaBi) or in a disaggregated database (such as ecoinvent), which is a crippling limitation.

Thus, a central motivation for this research is the aspiration of having comparable, consistent, and specific LCAs for all products. It is essential to recognize that LCAs need to move beyond being a niche practice if they are to have a substantial influence on the world. To shape taxation policies and consumer choices in a meaningful and fair way, LCAs must be reliably applied to a wide range of competing products, avoiding generic assessments that obscure the key differences between value chains.

### The need for a framework facilitating calculations of cradle-to-gate footprints

To achieve specific LCAs for a majority of products in the economy, there is a need for an efficient and scalable method leveraging primary data, without requiring companies to disclose their primary data. This requires fundamentally changing the way inventory and impact data are collected, exchanged, and stored.

### Research objectives and scope

The primary research objective is to develop the Specific SEmiautomated Lifecycle Footprinting (SSELF) framework, designed to facilitate semiautomated and value chain–specific cradle-to-gate product footprint calculations without requiring the exchange of sensitive proprietary data. This will allow us to achieve the secondary objective: identifying the potential challenges that need to be addressed to implement such a framework and the technical and societal implications.

Although the SSELF framework can be applied to any environmental impact category, the present focus is on carbon footprints. Carbon footprinting serves as a pertinent and timely example to guide readers through the development of the framework, illustrating its practical application and computational feasibility. This decision is informed by the growing demand for carbon footprint and greenhouse gas (GHG) emission reporting, driven by both regulatory requirements and voluntary corporate sustainability initiatives.

The scope of research is confined to attributional cradle-to-gate footprints, reflecting the point of sale perspective where use phase and end-of-life impacts are prospective and demand alternative estimation approaches. The focus of this paper is on the exchange of data between companies (data providers). While the intricacies of inner-company data management are not the focus of this study, they are briefly addressed in Section 5.

We conceptually develop the SSELF framework by reviewing existing initiatives and frameworks that strive to resolve the issue of data availability in LCA, building on their strengths and mitigating their weaknesses. We also explore the challenges that have to be addressed to achieve the goal of having specific footprints on a large scale. Last, we simulate the SSELF framework using Python to evaluate the computational effort as a function of the number of companies participating.

## DEVELOPING THE CONCEPTUAL FRAMEWORK

### Why we lack specificity in our current methods

There are several factors that contribute to the lack of specificity and scalability of LCA. Achieving specificity today requires the practitioner to deal with several obstacles (Ciroth et al., 2019; Miah et al., 2018):

1. Limited time and resources to collect primary data: An LCA routinely requires months to perform, and will often have to be outsourced to consultancy firms. The data collection phase is responsible for the majority of the time spent on an LCA (Miah et al., 2018), and this increases for each additional process or supply chain actor that has to be analyzed with primary data. Therefore, most LCA studies only use primary data for the foreground system that represents the company being assessed or the company producing what is being assessed.
2. Data confidentiality concerns: Even if the LCA practitioner had an unlimited budget of hours to collect primary data from the rest of the supply chain, they would likely struggle getting companies to hand over the necessary data to them. For most companies, such data contain confidential information on suppliers and bills-of-materials, allowing others to backcalculate profit margins, and more. Moreover, the data collection process routinely requires lengthy data privacy discussions and non-disclosure agreement drafting just to get permission to collect it, if at all.
3. The dynamic nature of supply chains and company data: The purchases, emissions, and sales of a company are not constant from one period to the next. Circumstances change continuously within the company, as well as upstream in their supply chain. The most common practice in LCA is to aim for data representing an annual average (Edelen & Ingwersen, 2016). This means that the LCI and LCA results are potentially obsolete within a year, and LCI has to be re-collected annually to ensure temporal representativeness.
4. High costs associated with LCA projects: Due to the time-consuming and complex task of current LCA studies, and the three challenges mentioned above, they are often a costly endeavor with little to no return on investment for the LCA commissioning company taking all the costs. This becomes a big blocking point of making LCA mainstream.

As a result, it is impracticable to calculate environmental footprints with specificity using our current data collection practices, and certainly to do this at a large scale for a high number of products and services. Therefore, consumers who wish to discriminate products and services based on their environmental impacts today are left to navigate a complex array of environmental labels of varying reliability, or to consult scientific literature on market averages (Brécard, 2017).

### Existing initiatives attempting to improve LCA availability and specificity

Several initiatives are proposing ways to tackle the issue of specificity and lack of coverage in LCA, particularly when it comes to GHG emission data and carbon footprints. Some of them focus on lowering the time and price barrier of conventional LCA through streamlining or automating the LCA process, others put novel technologies like Internet-of-Things (IoT) and Blockchain, or developing a new digital emission data exchange infrastructure, as potential solutions.

#### Adapting our LCA practices

Streamlined LCA emerged as a response to the high cost, time, and effort associated with conventional LCAs. The goal of streamlined LCAs is to simplify the LCA methodology and process to make it more efficient, less resource-intensive, and easier to perform, while still providing useful and meaningful results (Graedel, 1998; Gradin & Björklund, 2021). However, one must make compromises on the data quality, data uncertainty, and impact assessment, which reduces the accuracy of the results even further than conventional LCAs, providing merely proxies with a high cut-off and truncation or simply qualitative comparisons. Although it is easier to perform on a large scale than a conventional LCA, the specificity aspect is not addressed.

Automated LCA is another trend that can be observed in industry and research, gaining popularity due to its potential to reduce the manual workload associated with the LCI phase (de Oliveira et al., 2022). Data collection and analysis are partly automated by the digitization of production sites using industry 4.0 technologies, such as building information modeling systems, the International Material Data System, and enterprise resource planning (ERP) systems (Ferrari et al., 2021; Hollberg & Ruth, 2016; Haun et al., 2022). However, the automation is so far restricted to the company's own facilities; thus, it does not address the challenges of data collection from the background system (challenge 1 in Section 5.1) and is mainly relevant if one wants to frequently update one's own foreground data with minimal effort.

A number of private corporations and start-up initiatives aiming to tackle the aforementioned challenges are also emerging, such as Makersite (Makersite, 2024), Made2Flow (Made2Flow, 2024), Earthster (Earthster, 2024), and CarbonGraph (CarbonGraph, 2024), to name a few. These softwares aim to make LCAs more dynamic, scalable, automated (leveraging AI), and accessible to non-experts. This is in line with our research objectives as well; however, we advocate for a fundamental transformation in how we deal with and exchange data. The subsequent sections of this paper will highlight the necessity for a systemic transformation to address challenges that lie beyond the influence of individual corporate entities, advocating for an integrated framework that promotes efficiency at a large scale.

Moreover, while Environmental Product Declarations (EPDs) have become popular for communicating the environmental performance of products, these assessments suffer from the same fundamental challenges of LCA. This includes uncertainty and subjectivity in the selection of proxies and inconsistencies and data gaps in the proxy datasets themselves, since their compilation too was subject to challenges 1–4, compounding these problems and limiting comparability. Moreover, the EPD system lacks the necessary responsiveness to rapidly changing supply chains and market conditions. EPD databases, while offering more specific footprint data compared to broader LCA databases, are confronted with the high costs of creation and renewal, reliance on secondary data, and variability in category rules. These issues, combined with limited availability and awareness, highlight the need for systemic change to make LCA both efficient and accessible at scale (Ibáñez-Forés et al., 2016).

#### Making harmonized LCA part of regulations

Led by the European Commission, the product environmental footprint (PEF) methodology has been in development for several years (Manfredi et al., 2012; European Commission, 2013). The PEF aims to tackle the confusing and misleading maze of environmental labels and performance schemes by offering a reliable comparison metric for product environmental impacts. By establishing category rules (PEFCR) and harmonizing background data, it can be used to compare the environmental performance of specific products across 14 impact categories. However, although there has been an increased emphasis on primary data in this initiative, PEF still relies on generic data for many background processes. Despite efforts to harmonize the use of background datasets, the datasets come from a variety of LCA databases, leading to a risk of inconsistent modeling. Through a mid-term review led by the Commission, it was found that the method is *not* able to guarantee fair comparability (Lehmann et al., 2016). Definitions of product categories and representative products, as well as fixed generic datasets for background processes, are highlighted as potential causes of biases. When reproducibility over flexibility is preferred, this can lead to biased results that do not account for the real variations in upstream impacts. By restricting users to a rigid list of secondary datasets, greener products can end up being punished while less green products are rewarded.

#### Facilitating emission data exchange in supply chains

The use of traceability technology is being explored in LCA to help overcome the data collection challenges across supply chains. Blockchain-based LCA has very recently emerged as an area of research in which Blockchain is used in combination with IoT to collect tamper-proof LCI in a more automated way (Figueiredo et al., 2022; Köhler et al., 2022; Queiroz et al., 2019; Wang et al., 2018; Wu et al., 2022; Zhang et al., 2020), extending beyond the foreground system unlike automated LCA. Blockchain is a decentralized way of maintaining a digital record of events, minimizing the need for intermediaries and the risk of data manipulations, downtime, frauds, and hacks (Bahga & Madisetti, 2016). The challenges and obstacles of blockchain-based LCA include the increased cost, data processing and storage requirements, technology integration, energy consumption, governance challenges, and hardware requirements (Köhler et al., 2022; Liu et al., 2019; Tišma & Škrtić, 2023; Zhang et al., 2020). If the cost of doing a conventional LCA is too high for a company, blockchain-based LCA might be even further out of their reach. While blockchain and traceability may enhance LCA with secure and automated data capture, their high costs and technical demands limit their application and pose challenges to widespread adoption. Moreover, they are likely to be applied to only a restricted number of elementary processes within the product system because they will inevitably run into challenge 2 listed above; therefore, they do not fundamentally alter the dynamics that prevent efficient primary data collection.

Another initiative, this one more industry-led, is the Pathfinder initiative by the Partnership for Carbon Transparency (PACT). PACT's objective is to facilitate the exchange of product-level carbon footprints in a uniform and reliable manner to ensure more reliable, high-quality, and applicable data. To achieve transparency, PACT envisions emissions data traversing the value chain through an open, global network of interoperable software solutions for secure peer-to-peer exchange of product emissions data (WBCSD, 2021), with a focus on increasing the share of primary data. PACT references established product carbon footprint calculation standards and frameworks to create a more navigable hierarchy and offer more consistent guidance to practitioners.

Undoubtedly, PACT's initiative aligns closely with our research objective of providing widespread access to product carbon footprints with primary data for greater specificity. However, a challenge remains in semi-automating the assessment by linking production functions and unique product IDs to footprint calculations, rather than relying on manual, traditional methods in which the calculation and the data exchange are separate. Furthermore, the reliance on decentralized data exchanges rather than a centralized footprint database may lead to delays in updating footprints with the latest primary data from across value chains, as will be discussed in Section 4. The ripple effect of delayed data updates becomes more pronounced upstream—a bike producer calculating its 2022 carbon footprint might use 2021 data from the frame manufacturer, who used 2020 data from the steel supplier, who in turn used 2019 data from the mining company, thus leading to a compounded time lag. An automated system for real-time updates as suppliers revise their data would ensure that carbon footprints use temporally representative data for each supply chain entity.

## RESULTING FEATURES OF THE CONCEPTUAL FRAMEWORK

### Framework features required to solve issues hindering specificity and broad applicability in LCA

**FIGURE 2:**
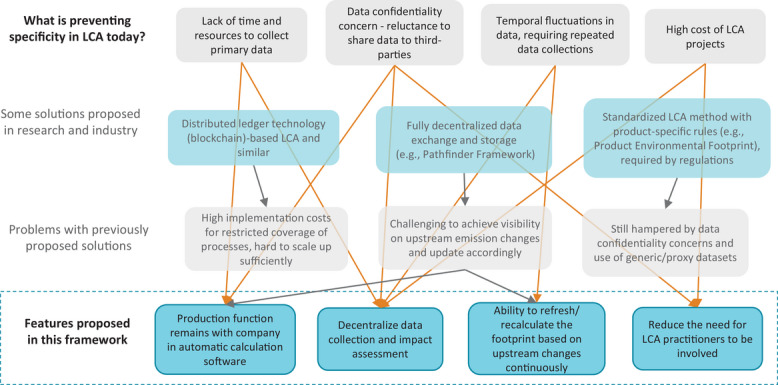
Summary of proposed framework features as a result of current challenges in life cycle assessment (LCA).

From the challenges that are preventing large-scale specificity in LCA, as well as reviewing the issues in existing initiatives attempting to address them, we have axiomatically derived features that need to be present in a framework to solve these, which are summarized in Figure 2 and described below.

To tackle the data confidentiality issue, several features must be present in the SSELF framework. Disclosing production data and suppliers is a significant blocking point, even if to a closed database. Moreover, if all companies were to disclose this to the same database, that would bring concerns on data privacy and security and possibly lead to reduced trust and a lower participation rate. In other words, data collection and footprint calculation need to be decentralized so that companies are only required to disclose the final aggregated footprint values. This disclosure also has to be done in a manner that prevents reverse engineering or back-calculating of their production functions (i.e., their cost structures; see Table 1 for glossary of key terms). Therefore, production functions remain with the respective companies. This also means that matrix inversion is not possible, since there is no all-encompassing A-matrix to invert. An iterative calculation method has to be used instead. By storing annual production functions in an automatic calculation software (*without* this information leaving the company), this also helps to address the challenge of capturing temporal fluctuations, as each company only has to ensure their own data are up to date. Moreover, this framework proposes an open database of aggregated footprints and the ability to recalculate the footprint continuously as upstream impacts change. This is essential to get rid of proxies and ensures that footprints can capture all upstream impacts within few recalculations, and will be demonstrated in Section 4. Last, to achieve specific footprints of everything in the economy, the cost of such assessments need to be considerably lower than they are today. The combination of these features, as well as other potential possible (but not essential) features mentioned in the discussion, leads to a reduced need for LCA analysts and therefore also a reduced cost.

**TABLE 1:** Glossary table.

Term	Definition
Production function	A mathematical representation of the relationship between inputs (materials, labor, energy, etc.) used in the production process and the output (product or service) of a company.
Product UID	A unique identifier assigned to a product or service. This refers to a product type (batch, model, etc., depending on data granularity available) produced by a company in the reference period of reporting.
Direct emissions	Emissions from sources that are owned or controlled by the reporting entity, also known as Scope 1 emissions.
Indirect emissions	Emissions that occur as a consequence of the operations of the reporting entity, but occur at sources owned or controlled by another entity, including both Scope 2 and Scope 3 emissions.
Scope 3 emissions	Emissions from upstream and downstream activities in a company's value chain, including both the procurement of inputs and the use and disposal of its products.
Iterative calculation	A method of computing product footprints where the value is refined through multiple rounds of calculation as new data become available, ensuring the inclusion of all upstream impacts.

### Ideation of the SSELF framework—A simplified thought experiment

So how can calculations be automated if there is not an omniscient matrix or database of unit processes and data collection and footprint calculation are decentralized?

For now, let us temporarily put aside a certain number of practical issues, to be revisited in Section 5, to focus on the core issue. Therefore, let us assume that:

1. All companies participate in this framework;
2. Each company knows their annual direct GHG emissions;
3. Each company knows its annual bill of goods required in production (i.e., its production function), but of course, it does not wish to share this information;
4. No company cheats or lies (revisited in Section 5.1.5)

Also, to simplify the illustration, and without loss of generality (see Section 5.1.3), let us have that each company produces a single product.

**FIGURE 3:**
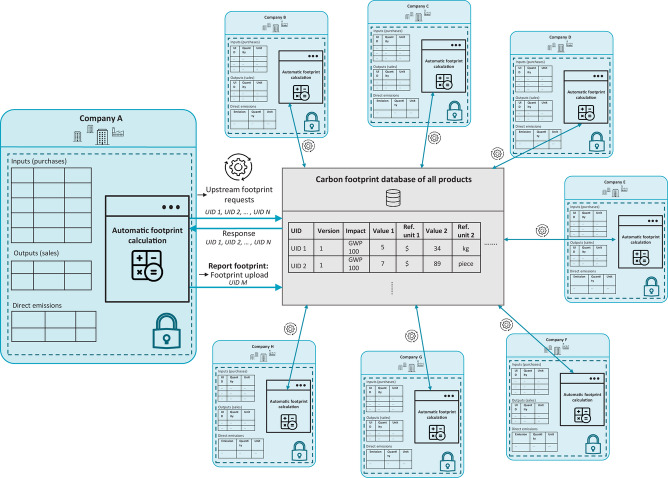
Conceptual figure showing the data exchange between companies and the footprint database. The lock icon highlights the fact that purchase data never leave the company (company A, in blue) but is used in calculating/updating product footprints, which are published for all (gray) in a database, and then used by other companies in updating their own footprint (companies B–H), and so on iterative (endless wheel icon). UID, unique identifier.

In this hypothetical scenario, how could we efficiently get specific carbon footprints (CFs) of everything while respecting corporate confidentiality concerns? To start with, we need to get rid of proxy choices: the footprints need to be calculated based on the actual, specific, inputs to the company, meaning there is a need for unique identifiers (UIDs) for products and services. Second, at any given time, we need to be able to instantly access the current, best estimate of the (aggregated) footprints of these purchased products, and thus the need for a public database of the latest calculated footprints. Third, we need primary company data to be the basis of the calculation but never exposed, which is possible by having a calculation software or tool where bills of goods can be entered for calculation purposes but never leaving the company's computer. To the fourth and last point, calculating and updating a product's footprint means multiplying the footprints of your inputs (per UID) by your own primary activity data on your purchases, plus your direct emissions. This is illustrated conceptually in Figure 3, where each company, by knowing their purchases, sales, and direct emissions, can use this to calculate their CF semi-automatically. For each of the UIDs of their purchases, a footprint data request is made to the database. With this, the company can calculate their footprint in the simplest of cases as shown in Equation 1, using monetary units as an example.

1 $${{\mathrm{Product\,CF\,per}}\$}_{\mathrm{Product\,A}} = \frac{\mathrm{Direct\,emissions} + \sum_{\mathrm{n} {\epsilon} \mathrm{purchases}} \mathrm{Purchase}_{n} \cdot \mathrm{CF\,per} \$_{n}}{\mathrm{Total\,sales}_{\mathrm{Product\,A}}}$$

**FIGURE 4:**
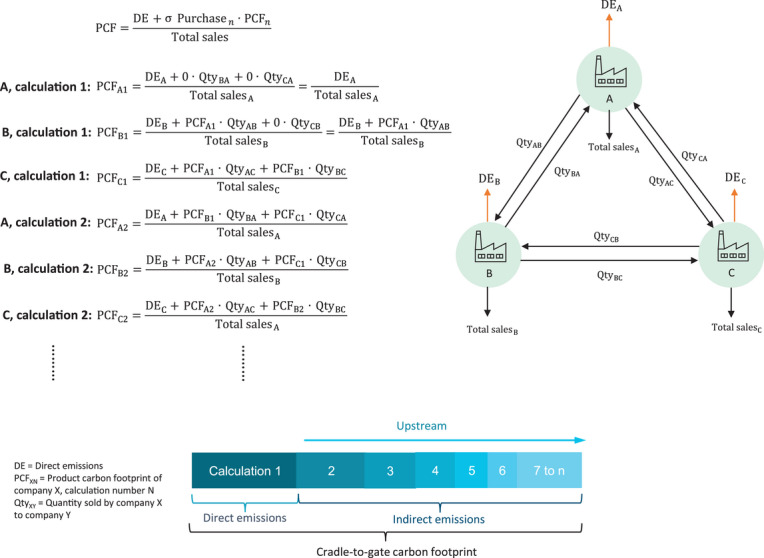
Illustration of how the footprints incrementally capture more and more upstream emissions as users update and report their footprints.

At the launch of the framework, all the footprints in the public database are zero; not a very realistic picture, but one that will iteratively improve very quickly. Let us imagine that company A is the first to apply equation 1 and report its product's footprint to the database. Since all other products have a footprint value of zero, the reported footprint of product A consists only of the direct emissions, as shown in Figure 4. However, as company A reports its new footprint to the database, the companies purchasing from company A get an automated warning that one or more of their inputs has an updated footprint in the database, and they automatically rerun Equation (1). They then automatically upload the recalculated footprint of their products to the database, after which company A receives a notice that one or more of its inputs has an updated footprint in the database, and so on. This happens iteratively through supply chains, resulting in each company updating several times as the footprints of their purchases gradually grow, until they eventually converge to a final value. The number of iterations required, or recalculations if you will, may depend on several factors, such as how significant the indirect emissions are to the total footprint. This is simulated using Python in the next section to see how many recalculations per company are required for the footprints to converge and to investigate if and how this number is affected by the number of participating companies and supply chain exchanges.

**FIGURE 5:**
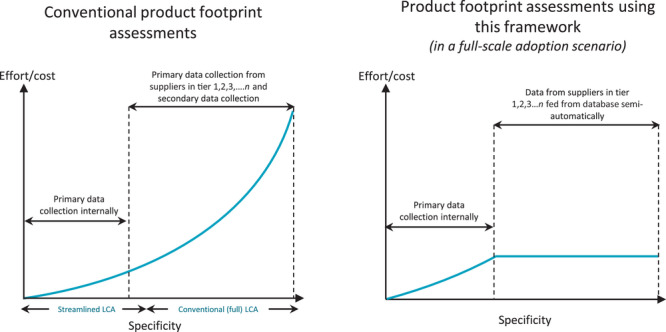
Illustration of how using the framework reduces the effort and cost of achieving specificity. LCA, life cycle assessment.

As a result of these features, the previously exponentially increasing effort required to increase LCA study specificity is non-existent using the SSELF framework in a full-scale adoption scenario, as illustrated in Figure 5. Each company would only need to collect and model their own foreground system (gate-to-gate), while the upstream impacts self-assemble through automated recalculations as other companies update theirs.

## SIMULATION OF THE SSELF FRAMEWORK

A simplified implementation of the SSELF framework was simulated using Python. The goal of simulating a simplified version of this framework was to investigate how computational requirements are affected by the number of participants and supply chain exchanges, to ensure that they do not increase in a way that makes its use impractical or infeasible on a large scale.

A central database for the publicly available footprint estimates for each product was set up as a simple table (as in Figure 3, in gray). A company class was then created, allowing the instantiation of multiple objects mimicking the behavior of individual companies. Each company-object holds as (private) attributes its list of annual purchases and sales, along with its direct emissions (as in Figure 3, in blue). Each company instance also has predefined methods for the following actions: downloading the latest estimate of the footprint of its purchases, calculating the footprint of its product (following Equation (1)), reporting the footprint to a database, and checking for the necessity to recalculate the footprint (if upstream footprints of its inputs have changed since the last calculated footprint was reported).

The footprint calculation is performed using Equation (1). The check_update_needed() function will compare the calculated footprint with the most recently published footprint and only lead to an update if the two are not equal or not close to equal, as determined by the isclose() function of Numpy (Harris et al., 2020) with default tolerances.

Exchanges between these virtual companies were set up, modeling various scenarios of supply chain exchanges, ranging from a scenario where all companies buy and sell to all companies except themselves (simulating a worst-case scenario) to where they buy and sell to 10% of companies chosen at random. To simplify simulations, but without loss of generality, each company produces only one product.

To have somewhat realistic numbers for GHG emissions per $ sold and profit margins, the Canadian physical flow account (Statistics Canada, 2023b) and supply and use tables (Statistics Canada, 2023a) for 2019 were used to extract their ranges. For the various sectors, GHG emissions ranged from 0–657.8 tons CO $$_{2}$$ eq. per million $ supply (certain sectors have no direct emissions, such as the “Office supplies” sector, only indirect emissions), and margins ranged from 28% –86%. The latter plays a factor in the number of recalculations required per company, because, looking back at Equation (1), if the denominator (sales) increases while the numerator (purchases and emissions) remains the same, the contribution of the numerator to the footprint becomes less significant. Therefore, the isclose() comparison will be satisfied sooner, resulting in fewer recalculations per company. The GHG emissions and margin per company are set to a random number within these respective ranges.

As previously mentioned, when the first company calculates and reports its footprint to the database, it includes only their direct (scope 1) emissions, since their purchases' footprints are not reported yet, and in this simplified Python simulation, they are therefore zero. With more companies calculating and reporting their footprints, everyone's upstream emissions incrementally increase with each recalculation, as more of their upstream emissions become included.

**FIGURE 6:**
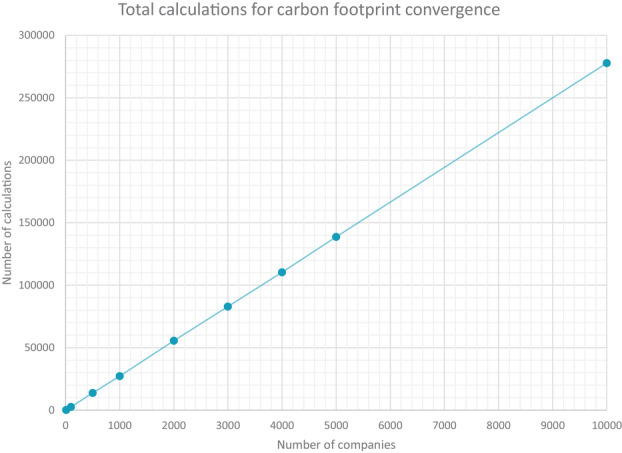
Simulation in Python of the number of calculations required for footprints to converge as a function of the system size. Underlying data are available in Table 1 of Supporting Information S1.

Figure 6 shows the total number of times companies need to automatically recalculate and report in order for the footprints to converge to a final value. It was found that the number of supply chain exchanges (i.e., whether everyone purchases from everyone or only from 10% of companies) did not affect the results; thus, Figure 6 shows the results for both scenarios. As can be seen from the graph, it is almost perfectly linear, which is the key takeaway from this simulation. With the variables used for this simulation, the average number of calculations per company remains nearly constant at 26–28 regardless of system size, with minor fluctuations occurring only because of the randomness of some variables causing them to change from one simulation to the next. Although the exact number of recalculations will be influenced by the structure of the real economy, this simulation shows that the computational requirements do not increase prohibitively with the size of the system; rather, they grow linearly with the number of companies participating. That being said, it is reasonable to conclude that the number of recalculations (“refreshing” the footprint) each company has to do to capture all upstream impacts through this method can be expected to be in the range of tens, and not hundreds or thousands.

## DISCUSSION

### SSELF framework implementation challenges

This section will go through the potential challenges encountered in trying to achieve widespread specificity of product footprints through the use of the SSELF framework.

#### Generating, communicating, and coordination of UIDs along value chains

The systematic use of UIDs for products and services is essential for semiautomated footprint calculations. In this context, UIDs uniquely identify goods and services, ensuring that each instance with the same UID shares the same footprint value. While UIDs for products are not new, in this context they must change when a product moves from one company to another and its footprint changes. For instance, the apples sold by a farmer to a wholesaler require a different UID than the same apples sold later by the wholesaler to a grocery store, since the apples will have a different footprint after the transport, storing, and cooling that occurred after being passed to the wholesaler.

There is no single, global identifier system, but several standards do exist. For instance, a Global Trade Item Number (GTIN) typically identifies a product type and remains constant when transferred between companies (GS1, 2017). In contrast, a Universally Unique Identifier (UUID) uniquely identifies specific objects or records, like individual product instances or transactions, and new owners may generate a new UUID for their tracking purposes. For the purposes of this framework, there needs to be a consistent and appropriate system used for product identification, in which established standards like those in the GS1 Global Traceability Standard, which emphasizes unique product identifiers, data consistency, and global compatibility (GS1, 2017), will be useful. As no single, global system exists, the framework should be flexible and accept different identifier systems.

#### Capital goods and other nonconsumables

In many cases, annual inputs to a company may not accurately represent average annual flows to a product system. For long-lasting goods like buildings or equipment, it is more appropriate to amortize their impacts based on expected lifespans and production volumes. However, this approach often lacks precision as lifespan estimates are typically generic averages for the commodity type. Estimating lifespans can also be subjective due to varying references. To reduce subjectivity and automate this part of footprint assessment, embedding it in software is beneficial. If all purchases, including capital goods, have a UID, lifespans can be included as an attribute of the product in the SSELF framework, allowing the software to automatically amortize a portion of the capital good to the relevant product function(s). The remainder of the capital good is “transferred” to the next year's inputs until all of its impacts have been accounted for. However, this approach will not cover purchases made before implementing this framework, as they lack UIDs or footprint data in the database, in which case their impact should be treated as data gaps, as explained in Section 5.1.8, but with a similar logic otherwise.

#### Disaggregating corporate inventories to products

As many companies produce more than one product or service, some even in tens of thousands, they should ideally disaggregate their corporate inventories to their outputs in a manner that reflects the reality as closely as possible with the available time and data. Many companies have partially joint productions, meaning that some inputs and emissions are possible to subdivide to specific products, and others are common to many products and not possible to subdivide without the use of allocation. This can be achieved by users specifying which and how much of purchases are tied to the various production outputs (production functions) using bills of materials (BOMs), recipes, and similar data. For example, they might know how much aluminum from supplier X went into bike Y, while bike Z used aluminum from a different supplier. Similarly, direct emissions from a company can be disaggregated or allocated to production outputs. Existing sector- and product-specific rules can guide these disaggregation decisions. How one deals with multifunctionality and disaggregation may significantly impact a product's footprint and be used to gain a competitive advantage. Guidelines such as PEFCRs, ecoinvent system models, or Life Cycle Initiative's Global Guidance Principles for Life Cycle Assessment Databases can help ensure consistency within product systems and can even be integrated into the framework based on the product or sector type (WBCSD, 2021; Sonnemann et al., 2013; Wernet et al., 2016). That being said, having a process-based LCA inventory for each and every product given real world challenges and constraints is likely unrealistic, and even by following the aforementioned guidances and system models, comparisons across product categories remains a challenge due to inconsistencies across product category rules. This highlights the importance of product category-agnostic guidance principles if we want an objective footprinting system that can be used to compare footprints across different categories of products.

A generic formula for calculating the specific footprint of a product or service, assuming monetary units for simplicity, is shown in Equation (2) below. Carbon footprint is used as an example, but the logic applies to any impact category. General company inventory remaining after these disaggregations can be allocated to all sales, here using economic allocation as an example, as this would typically be the most straightforward allocation method to apply at a large scale. This approach keeps system boundaries complete (from a corporate perspective) and ensures that all corporate impacts are taken into account.

2 $$\mathrm{Product\,CF\,per} \$_{\mathrm{Product\,A}} \;=\; \frac{\mathrm{Direct\,emissions}_{\mathrm{Product\,A}} + \sum _{\mathrm{n} \epsilon \mathrm{specific\,purchases}} \mathrm{Purchase}_{n} \cdot \mathrm{CF\,per} \$_{n}}{\mathrm{Total\,sales}_{\mathrm{Product\,A}}} \nonumber\\ \;+\; \frac{\mathrm{Direct\,emissions}_{\mathrm{residual}} + \sum _{\mathrm{m} \epsilon \mathrm{residual\,purchases}} \mathrm{Purchase}_{m} \cdot \mathrm{CF\,per}\, \$_{m}}{\mathrm{Total\,sales}_{\mathrm{all\,production\,units}}}$$

#### Harmonization through automation

Achieving broader, more specific and semiautomated footprints necessitates reducing the reliance on analysts as intermediaries. This requires automating complex and expertise-dependent tasks in footprint assessments. Fortunately, many challenges typically requiring skilled analysts, like defining system boundaries, selecting proxy datasets, and navigating background databases, become non-issues in this system. The remaining methodological concerns are multifunctionality and choosing reference units or functional units, the former being discussed briefly in Section 5.1.3.

Automating parts of the carbon footprint assessment not only saves time and costs but may also contribute to harmonization if performed in a suitable way. Methodological inconsistency is a known issue in the community, with manual approaches often yielding varied results due to subjective interpretation and assumptions. If done correctly, automation can reduce the risk of inconsistency and subjectivity.

#### Credibility and verification

The integrity of any reporting framework hinges on the accuracy and reliability of its reported outputs. The challenge is even greater for the SSELF framework, since it aims to minimize the need for direct involvement of LCA analysts. To maintain the credibility of the reported footprints, a combination of top-down and bottom-up measures can be employed.

Since the SSELF framework can be used to keep track of and report scope 1 (direct) emissions alongside product footprints, this allows for cross-verification of sector-wise direct emissions with those in national GHG emissions inventories.

Moreover, benchmarking the product footprints against existing LCA and EEIO databases can serve as a heuristic for detecting significant anomalies that may suggest inaccuracies or fraudulent reporting. Eventually, once the SSELF framework has sufficient coverage within product categories, this database can also be used to detect anomalies. Such discrepancies, if found, would warrant further investigation. Audits, both randomized and targeted, can further enhance verification by checking that the sum of bills of goods entered as production functions matches the expenses declared for tax purposes. Companies in most jurisdictions have long had to declare sales and purchases for tax reasons, and increasingly their scope 1 emissions must also be reported and can be subject to audits. The proposed process of entering their BOMs in the footprint calculator of Figure 3 can be seen as the next step in the auditable declaration requirements of companies. In fact, all of these different reporting processes could be combined for higher efficiency and to ensure consistency.

It is important to note, however, that this article does not aim to define a definitive verification process or business model to deter non-compliance. While we outline potential measures that can enhance reliability, it is understood that no system is infallible, and a well-defined business model to ensure data integrity will be crucial. Given the current challenges associated with scope 3 emissions reporting—often characterized by unreliability and incompleteness (Hettler & Graf-Vlachy, 2024; Nguyen et al., 2023)—we believe that the SSELF framework, supplemented by the verification measures suggested above, would substantially improve the dependability of footprint reporting.

#### Incentivizing participation

The SSELF framework can only achieve complete and fully value-chain-specific footprints if all companies participate, since they are also the data providers. Each non-participating company leads to a data gap that forces the use of generic data, with its associated challenges with respect to consistency. A spontaneous or voluntary emergence of a network for the SSELF framework, driven by business models alone, is unlikely. Mandatory participation through regulations will likely be necessary to reach a critical threshold of involvement across businesses within a given geography. This would ensure the network's viability and the framework's effectiveness from the outset.

Regulatory pressure is increasingly becoming a driver for environmental reporting, particularly for GHG emissions. Many jurisdictions are introducing legislature that mandates corporate GHG emission disclosures, with some extending requirements to include scope 3 emissions. The Corporate Sustainability Reporting Directive (CSRD) require all large companies and listed companies in the EU to assess and disclose their environmental impact, in which carbon data are a crucial reporting element (European Commission, 2022). As the United States prepares for GHG emission reporting legislations, a survey by PWC found that 61% of business executives anticipate costs exceeding $750,000 in the first year of compliance and 85% express concerns about the lack of suitable technology (Workiva & PwC, 2023). Furthermore, the EU recently adopted a regulation requiring product-level carbon footprint assessments for certain goods being imported to the region to ensure equivalent carbon pricing for imports and domestic products, called the Carbon Border Adjustment Mechanism (CBAM) (European Commission, 2023). Should producers choose not to measure or calculate their own carbon footprints, they may use default values, potentially leading to a higher carbon tax. Moreover, we are seeing the introduction of tax incentives such as the Hydrogen Production Tax Credit, which is part of the Inflation Reduction Act in the United States. Producers can get tax credits if their well-to-gate GHG emissions are less than 4 kg CO2eq/H2 (Office of Energy Efficiency & Renewable Energy, 2024).

Beyond regulatory pressure, the framework's ability to provide more specific footprints at a lower effort than today's methods, as shown in Figure 5, can serve as a competitive advantage for businesses, encouraging involvement. However, as mentioned, due to the nature of this framework hinging on a high participation rate, the ideal scenario is a direct legislation mandating GHG or footprint calculations and disclosure through the use of this specific framework. Should participation be entirely voluntary, then the success of this framework largely depends on the trust in the quality and accuracy of the data reported.

#### Managing the database

Another question that arises in the implementation of this framework is who would be responsible for managing and overseeing the footprint database. Although this question is not a focus of this article, it is worth mentioning that there are many options, each with its advantages and disadvantages: A government agency would offer regulatory authority, an international organization can ensure global harmonization, industry consortia would enable sector-specific expertise, nonprofits can provide independent oversight, and public-private partnerships might combine diverse strengths. The choice would depend on factors such as regulations, technological feasibility, and industry cooperation.

#### Dealing with background data gaps

In an ideal world, every product and service would have their footprints in the database, eliminating data gaps (as simulated in Python). However, in the real world, this is unlikely due to complex, dynamic, and globalized value chains with varying maturity and motivation for data collection. Moreover, in an entirely voluntary participation scenario, it will be necessary to deal with data gaps to ensure that the framework does not provide artificially low results due to lack of footprint data. Although these measures can bridge temporary gaps, they are still stopgap solutions that do not fully harness the potential of the framework.

To address potential data gaps, data from other sources can be used as proxies. Taking inspiration from CBAM, default emission factors (representing world and country averages) by type of goods can be used in a transitional phase, ensuring that the calculated footprints can still be useful during this phase. Eventually, when there is a high quantity of footprint data available through the framework, we can move away from LCA/EEIO proxies and instead leverage the data in the framework for this.

While dealing with data gaps is not a new challenge, placing this responsibility on non-experts within companies highlights the need for a more automated and consistent approach, ensuring that the framework can produce valuable results even if not everyone participates. Machine learning can be a part of this solution, by allowing users to input data and metadata about a product or service (type, origin, units) and suggesting the most suitable proxy datasets. Tools such as CaML and Flamingo already exist for this purpose, automating the selection of appropriate EEIO or LCA datasets through semantic text similarity matching, leveraging product and industry sector descriptions. Although not perfect, these tools eliminate the manual labor typically required and achieve good accuracy, with a 75% accuracy rate for LCA predictions (Balaji et al., 2023) and a mean absolute prediction error of 22% for EEIO predictions (Balaji et al., 2023). Integrating this functionality into the tool illustrated in Figure 3, alongside databases for proxy data, offers a more consistent, objective, and automated approach to address data gaps.

### Potential implications of implementing the framework

The adoption of a framework enabling every product and service to have a specific footprint label carries many positive implications. First, democratizing LCA provides accessible and standardized environmental impact information for products, empowering consumers to make informed, sustainable choices.

Additionally, this framework reduces the effort required for corporations to conduct specific and precise upstream scope 3 assessments as well as product footprint assessments, as shown in Figure 5. Upstream scope 3 emissions, covering indirect emissions from a company's value chain, often pose challenges in data collection and calculation. The proposed framework significantly eases the burden for companies in achieving specificity in their scope 3 assessments. This can encourage broader adoption of comprehensive accounting and reporting practices on GHG, leading to better informed decision-making and more effective emissions reduction initiatives.

Furthermore, the framework offers governments valuable insights for designing effective environmental policies, such as carbon taxes. It enables policymakers to develop targeted interventions that incentivize emission reductions across sectors and encourage the transition to a low-carbon economy. As previously mentioned, EU's CBAM attempts to encourage cleaner production in non-EU countries through a carbon tax (European Commission, 2023). However, the mechanism's scope is limited to only a few types of basic goods, since it is overly burdensome to ask producers to assess the carbon footprints of all carbon-intensive finished goods, particularly those with complex supply chains. Therefore, the CBAM is currently applied to a limited number of intermediary products (e.g., cement, iron, steel, aluminum, fertilizers, electricity, and hydrogen) and very few finished products. This may inadvertently incentivize the relocation of manufacturing for finished products to non-EU countries to circumvent the carbon tax. Implementing this framework to deal with carbon tax at a product-level would provide comprehensive coverage, eliminating such risks.

However, implementing the SSELF framework for carbon footprints alone may shift environmental burdens to other impact categories beyond climate change. While the focus of this paper is on carbon, it is important to consider the broader environmental impacts of products and services, such as water scarcity, ecotoxicity, or land use. Applying the framework logic to other impact categories or endpoint-level impacts can mitigate this risk.

### Strengths and limitations

Apart from the positive implications discussed in the previous section, a key strength of this framework is that it would provide valuable and mass-customized insights into cradle-to-gate environmental impacts. In addition to the value this would have for companies and consumers, it could serve as a valuable resource for LCA practitioners by complementing or replacing the rather generic datasets available in databases, offering enhanced specificity in their assessments. By incorporating data on the variability of footprints within specific product categories, these databases can offer improved generic datasets that take advantage of the massive amount of footprint data from the framework, albeit in an aggregated format.

Although the SSELF framework shares commonalities with systems such as EPDs, where primary data are increasingly leveraged, it aims to address the inherent inefficiencies and slow responsiveness associated with current practices. Unlike EPDs, which may still exhibit inconsistencies due to variable system boundaries across product categories, the SSELF framework envisions a more streamlined and uniform approach that can potentially eliminate the need for system boundaries altogether. This approach enhances the consistency and comparability of LCA data across products. While the costs associated with producing and updating EPDs pose a barrier to realizing LCAs for all products, we propose that by rethinking data exchange and storage practices, we can mitigate these challenges. The SSELF framework offers an alternative that, with strategic modifications, can facilitate the broader application of LCAs. That being said, as mentioned in Section 5.1, challenges beyond the influence of a framework on its own remain to achieve the end goal of having LCAs for all products.

Another benefit of using the SSELF framework for footprint calculations is that LCIA regionalization becomes easier. Since the potential environmental impact of an emission or extraction depends on where it occurs, sometimes affecting its impact by several orders of magnitude, it is important to consider this in the impact assessment (Huijbregts & Seppälä, 2000; Humbert et al., 2009; Rosenbaum et al., 2007). Knowing where an elementary flow occurs can be a major challenge for an LCA practitioner, particularly for the background system. When each participant simply has to know where their own flows occur, this allows for more detailed modeling. Although such regionalization is not relevant in carbon footprint assessments, it is an important consideration for other impact categories, and something this framework can achieve with significantly better spatial precision.

Lastly, government agencies can find value in this framework. It aids in understanding the variability within product systems that deliver the same service. This knowledge can inform policy development and regulatory decisions related to carbon emissions and sustainability.

A limitation of the SSELF framework is that it does not include the gate-to-grave phases encompassing product use and end-of-life impacts. While cradle-to-gate assessments provide valuable insights into the upstream burdens, the exclusion of the downstream presents a gap in understanding the complete life cycle impacts. The use phase often contributes significantly to the overall footprint and should be considered for a fair and holistic product comparison. However, these downstream impacts are yet to occur at the point of purchase. Estimating gate-to-grave impacts requires a different approach and additional data and assumptions beyond supply chain exchanges and emissions. Further research is needed to enhance the specificity of these estimates and incorporate them into a semiautomated calculation.

Another limitation of the SSELF framework is the lack of transparency regarding where impacts occur throughout the supply chain, since the result of each calculation is an aggregated total footprint. This makes it difficult to conduct detailed hotspot analyses. This limitation restricts the framework's utility for ecodesign, as it prevents the investigation of specific reasons behind varying footprint levels. However, this trade-off may be necessary to respect company confidentiality while promoting widespread adoption of specific LCAs. Despite this, aggregated footprint scores still provide valuable metrics for guiding consumers toward environmentally friendly options and impact reporting. For those aiming to perform hotspot analyses and investigate improvement potentials, less aggregated databases can be utilized. Additionally, companies using the SSELF framework can still perform contribution analyses with visibility into the aggregated impacts of their inputs, allowing for some degree of ecodesign optimization.

This framework is designed for attributional assessments, which some LCA experts argue may be less suited if the goal is to influence decision-making processes, in which case they believe a consequential perspective should be favored (Weidema et al., 2018). Ultimately, attributional and consequential LCA respond to two different questions and are both necessary to manage environmental responsibility (Brander et al., 2019). Attributional LCA attempts to quantify the potential global impact share of a product life cycle, while consequential LCA attempts to quantify the potential impacts that occur as a consequence of a decision (Ekvall, 2019; Finnveden et al., 2009; Schaubroeck, 2023). A change in demand for a product based on its perceived environmental friendliness can have unintended consequences when viewed from a consequential LCA perspective. However, for retrospective analysis where the goal is to understand the past environmental burden of products, attributional LCA offers a more direct and practical approach, avoiding the complexities of modeling the broader economic interactions that consequential LCA seeks to capture for future-oriented assessments.

An important limitation of this framework is its dependence on a high rate of participation. The more companies that adopt it, the more primary data contribute to the footprints. The benefits of using it must outweigh the associated effort and cost. While individual companies may weigh the benefits against the effort and costs, the true value of the framework emerges when considering the collective gains. Efficiency improvements and comprehensive environmental insights, as depicted in Figure 5, underscore the advantage of the framework over traditional LCA assessments, particularly when specificity is paramount. Thus, it is the collective benefit that can drive regulatory bodies, influenced by investor and consumer demands for transparent GHG and LCA reporting, to mandate the adoption of the framework.

Lastly, there is also the risk of companies manipulating or providing misleading disclosures. While this framework aims to enhance transparency and accountability, the potential for companies to manipulate or misrepresent footprint data exists. Robust verification, auditing mechanisms, clear standards, and guidelines are essential to prevent greenwashing and maintain the integrity of reported footprints.

## CONCLUSION

Our exploration into the SSELF framework challenges the LCA community's traditional reliance on unit-process databases for background data. We have grown accustomed to the idea that the only way to calculate an LCA score is through a central authority's A-matrix. The SSELF framework presents an alternative—a method that compiles footprints through the accumulation of impacts along value chains, offering a significant departure from the norm.

This new method has the potential to address some of the most persistent challenges in our field, such as time constraints for data collection, data confidentiality concerns, high costs, inconsistent and incomplete system boundaries, and proxy selection. By shifting away from a central proxy-based database and embracing a value chain-based footprint accumulation, we can potentially overcome many of the most enduring challenges of the LCA community.

However, this novel approach does not come without its own set of challenges. This prompts us to reconsider how we manage product IDs, how we handle disaggregation, and how we approach the issue of multifunctionality. There are important considerations to be made when trying to go from separate process-based LCA studies to a scalable approach that can cover all products consistently. These are not minor adjustments but rather fundamental aspects that will require careful thought and further research.

In summary, the SSELF framework is an innovative step toward more specific and universal LCAs, potentially making environmental reporting easier and more cost-effective for companies. Yet its success is tied to how widely it is adopted and how trustworthy the data remain. Since companies are also the data providers, each non-participating company represents a data gap that forces the use of secondary, less specific, data. A spontaneous emergence of a network of participating companies is unlikely; thus, regulations are key to its viability.

## Supplementary Information

Data S1. Excel file containing simulation results. Table S1: Python simulation of the number of calculations required for all footprints to converge to a final value, as a function of the system size and the number of exchanges between companies. Table S2: Computation time for all footprints to converge as a function of the number of companies. (XLSX 15.1 KB)

## Data Availability

The data that support the findings of this study are derived from public domain resources: the Canadian physical flow account (Statistics Canada, 2023b) and supply and use tables (Statistics Canada, 2023a) for 2019. The specific data extracted from these sources are available in the Python script openly available in the Zenodo repository at https://zenodo.org/doi/10.5281/zenodo.12802860. The underlying data shown in Figure 6 are available within Supporting Information S1.
